# Effects of intermittent seating upright, lower body negative pressure, and exercise on functional tasks performance after head-down tilt bed rest

**DOI:** 10.3389/fphys.2024.1442239

**Published:** 2024-09-06

**Authors:** Gilles Clément, Sarah C. Moudy, Timothy R. Macaulay, Edwin Mulder, Scott J. Wood

**Affiliations:** ^1^ KBR, Houston, TX, United States; ^2^ Aegis Aerospace, Houston, TX, United States; ^3^ DLR, Institute of Aerospace Medicine, Cologne, Germany; ^4^ NASA Johnson Space Center, Houston, TX, United States

**Keywords:** sensorimotor system, vestibular tests, bed rest, spaceflight, countermeasure

## Abstract

**Introduction:**

Bed rest can be used as a ground-based analog of the body unloading associated with spaceflight. In this study, we determined how strict head-down tilt bed rest affects subjects’ performance of functional tests (sit-to-stand, tandem walk, walk-and-turn, dynamic posturography) that challenge astronauts’ balance control systems immediately after they return from space.

**Methods:**

Forty-seven participants were assessed before and a few hours after 30 days of 6° head down tilt bed rest at the DLR:envihab facility. During this bed rest study, called SANS-CM, the participants were divided into 4 groups that either a) were positioned in head-down tilt continuously throughout the 30 days; b) sat upright for 6 h a day; c) were exposed to lower body negative pressure (LBNP) for 6 h a day; or d) exercised for 60 min and then wore venous-occlusive cuffs for 6 h a day.

**Results:**

Results showed that strict head-down tilt bed rest caused deficits in performance of functional tasks that were similar to those observed in astronauts after spaceflight. Seated upright posture mitigated these deficits, whereas exercise or LBNP and cuffs partly mitigated them.

**Discussion:**

These data suggest that more direct, active sensorimotor-based countermeasures may be necessary to maintain preflight levels of functional performance after a long period of body unloading.

## Introduction

During space travel, astronauts experience microgravity, which causes the sensorimotor system to undergo significant reinterpretation of both body axial-loading and vestibular/proprioceptive information. When they return to Earth, astronauts encounter disturbances in perception, spatial orientation, posture, gait, eye-head coordination, and head-trunk coordination. Astronauts have demonstrated signs of reduced functional mobility after spaceflight, as assessed by their performance in an obstacle course (Clément et al., 2022a).

Bed rest can be used as a terrestrial analog for studying the effects of body unloading associated with spaceflight. Study participants undergo strict bed rest at a 6° head-down tilt (HDT) to replicate the fluid shift and axial-unloading experienced in microgravity. However, these individuals still perceive gravity along the medial-lateral and anterior-posterior axes through the vestibular organs during bed rest. Consequently, bed rest primarily unloads the vertical axis along the head-to-foot body axis, facilitating direct examination of the impacts of this axial unloading. Bed rest studies therefore allow researchers to model the effects of the somatosensory deconditioning during standing, static balance, and walking after spaceflight (Reschke et al., 2009).

The research presented in this study was a component of a broader investigation known as the spaceflight-associated neuro-ocular syndrome countermeasure (SANS-CM) study, which included 4 campaigns of 30-day bed rest at 6° head down tilt conducted in the: envihab facility at the German Aerospace Center in Cologne, Germany. The objective of the first 2 campaigns, performed between September 2021 and March 2022, was to determine whether daily 6-h exposures to lower body negative pressure (LBNP) or seated upright posture mitigated the deterioration of ocular anatomy and physiology, as well as musculoskeletal, cardiovascular, hematology, immunology, behavioral, and balance functions. The objective of the third and fourth campaigns, performed between January and June 2023, was to investigate how daily exercise and wearing thigh cuffs affects these measures, as compared to measures from a group of control subjects who did not use these countermeasures.

In this specific study, we analyzed how subjects performed functional tasks that test the balance control system both before and after 30 days of bed rest. We compared the performance of subjects who underwent strict HDT bed rest with and without specific countermeasures, aiming to investigate the impact of these countermeasures on individuals experiencing 30 days of axial body unloading. We hypothesized that the subjects who were not given countermeasures would experience greater impairments in balance and walking tasks after bed rest that the subjects who intermittently sat upright, were exposed to LBNP, or exercised and wore thigh cuffs.

## Methods

### Participants

The test procedures were approved by the institutional review boards of the National Aeronautics and Space and Administration (NASA) Johnson Space Center and the German Aerospace Center. Tests were performed in accordance with the ethical standards laid down in the 1964 Declaration of Helsinki. All subjects provided written informed consent before participating in the study.

**FIGURE 1:**
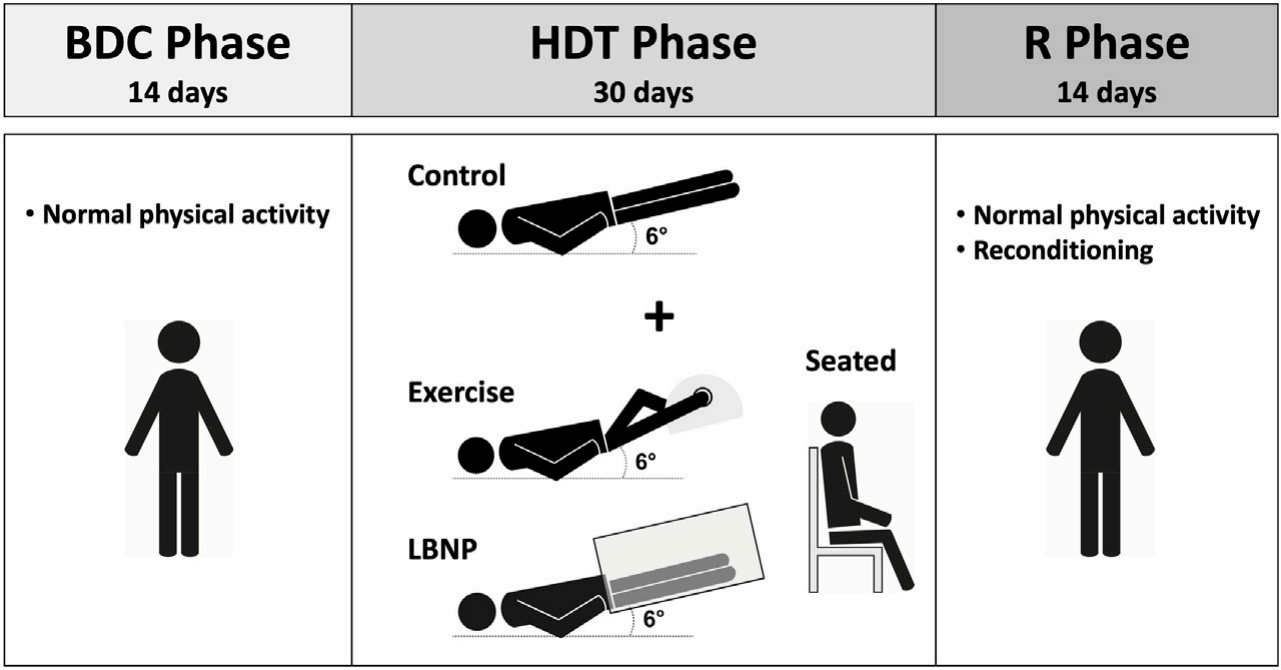
Schematic representation of the SANS-CM study design. BDC, baseline data collection; HDT, head down tilt; R, recovery.

The study comprised 4 groups of subjects, with mean anthropometrical values and standard deviations provided below:

(a) 12 participants (8 males, 4 females, aged 34.3 ± 8.9 years; height 1.75 ± 0.13 m; weight 73.5 ± 13.9 kg) who completed 30 days of strict, continuous bed rest at a 6° HDT.
(b) 12 participants (7 males, 5 females, aged 35.1 ± 8.5 years; height 1.74 ± 0.08 m; weight 69.6 ± 11.2 kg) who completed 30 days of strict bed rest at a 6° HDT and 60 min of aerobic cycle exercise 6 days per week in HDT position, after which they wore venous-occlusive thigh cuffs for 6 h.
(c) 12 participants (6 males, 6 females, aged 34.4 ± 10.3 years; height 1.72 ± 0.09 m; weight 73.4 ± 15.6 kg) who were exposed daily to 2 × 3 h of 25 mmHg LBNP during 30 days of strict bed rest at a 6° HDT.
(d) 11 participants (6 males, 5 females, aged 33.1 ± 6.0 years; height 1.75 ± 0.09 m; weight 71.6 ± 8.5 kg) who sat upright for 2 × 3 h a day during 30 days of strict bed rest at a 6° HDT (Figure 1).

The differences in age, height, and weight across these 4 groups were not significant (ANOVA single factor, *p* > 0.8).

### Countermeasures during bed rest

Prior to the study, all participants passed a medical examination and had no known history of vestibular or oculomotor abnormalities. Each subject resided for a total of 59 days (of which, 30 days were spent in HDT bed rest) in the:envihab facility at the German Aerospace Center in Cologne, Germany. Specifically, each subject underwent: a) a 15-day baseline data collection phase; b) a 30-day bed rest phase in HDT; and c) a 14-day recovery phase.

During the entire HDT phase the beds of all test subjects were tilted 6° head-down. Subjects did not use a pillow to support their head. Daily routine activities such as eating, washing, showering, toilet, and leisure activities (e.g., reading, watching TV) were performed in the 6° HDT position. To control the adherence to the 6° HDT position, the test subjects were instructed to touch the mattress with one shoulder at all times. Subjects were continuously supervised by dedicated staff via a closed video system that was also used to provide immediate feedback to the subjects. Test subjects were not allowed to raise, contract, or stretch their legs to minimize mechanical stimuli other than during physiotherapy.

Daily 60-min physiotherapy sessions were provided to bed rest subjects during both the HDT phase and the recovery phase as follows: a) from HDT1 to HDT10: massage of the upper and lower back, neck and shoulders; b) from HDT11 to HDT20: massage of lower back, legs and feet; c) from HDT21 to HDT30: massage of legs, fee, and lower back; and d) from the initial day upon return to upright (R + 0) to R + 10: massage of upper and lower back, neck and shoulders.

Within the first 3 days before the HDT phase, subjects in the exercise group completed 2 ramp tests on a cycle ergometer in the 6° HDT position. This ramp test was used to determine the appropriate work rates for use during the bed rest sessions. During this BDC phase, the intensity of the exercise bouts increased incrementally, so that subjects were completing the full 60 min of exercise by the start of HDT. During the HDT phase, subjects exercised for 60 min daily: 5 min of warm-up at ∼50% target wattage, 5 min of warm up at ∼75% target wattage; 45 min of exercise at target wattage; and 5 min of cool down at ∼50% target wattage. The subjects donned the venous-occlusive thigh cuffs within 30 min after the exercise concluded. The cuffs were adjusted to a pressure of 50 mmHg and they remained in place for 6 h. During this period, the pressure was monitored, and adjusted if needed, every 30 min.

During the HDT phase, the 2 daily sessions of 3 h in a seated upright posture took place once in the morning and once in the evening, mirroring the exposure times to LBNP. Subjects just sat in front of the desk near their bed with their feet in contact with the floor.

The third countermeasure consisted of 2 daily sessions of 3 h of LBNP at 25 mmHg during the HDT phase, also one in the morning and one in the evening. The duration of LBNP exposure was chosen due to logistic concerns of implementing 6 h of continuous LBNP. Subjects underwent a LBNP familiarization session before bed rest to allow them to get comfortable with the equipment and to make sure they fit well in the LBNP device.

### Functional tasks

Four tests were conducted before and after the HDT phase: sit-to-stand, walk-and-turn, tandem walk, and dynamic posturography. Participants of all 4 groups completed all tests in a randomized order. Test sessions took place on the day before the HDT phase and the day the HDT phase concluded. All subjects were familiarized with the tests and procedures 4 days before baseline testing. During these tests, the subjects wore a triaxial inertial measurement unit (IMU) (Opal V2 or Emerald, APDM Inc., Portland, OR, United States) attached to their trunk with an elastic band.

#### Sit-to-stand

This task involved subjects rising swiftly from a seated position without using their hands for assistance and maintaining a steady stance for 10 s. The task was repeated twice. The duration between the command to stand and achieving a stable posture served as the performance metric. The attainment of stable posture was determined using IMU data. The initiation and completion of the stand were identified by monitoring the absolute angular velocity of the trunk pitch (Clément et al., 2022a).

#### Walk-and-turn

After the sit-to-stand task described above, participants were instructed to walk swiftly and safely in a straight line toward a cone positioned 4 m away, circumvent the cone, return, and sit back in a chair. En route to and from the cone, participants traversed a 30-cm-high obstacle. This sequence was repeated twice. IMU data were used to calculate the time taken to navigate the walk-and-turn task and the maximum yaw angular velocity of the trunk during the turn around the cone (Clément et al., 2022a).

#### Tandem walk

Participants were directed to take 10 heel-to-toe steps with their arms folded across their chest and their eyes closed (2 trials) or open (2 trials). Each trial was recorded on video. Three independent reviewers analyzed the videos to ascertain the number of accurate steps in each trial. A step was considered a “misstep” if any of the following occurred: a) the stepping foot crossed over the planted foot, b) the subject veered to the side before completing the step, c) the stepping foot swung in a wide, arcing path before landing, d) the step duration exceeded 3 s, or I there was a gap larger than 10 cm between the heel of the front foot and the toe of the back foot after completing the step (Mulavara et al., 2018). To minimize reviewer bias, the order of the videos was randomized. The median value of all the reviewers’ assessments was used to determine the percentage of correct steps during each trial. A higher percentage of correct steps indicated better performance (Clément et al., 2022a).

#### Dynamic posturography

Postural stability was assessed using the sensory organization tests (SOTs) conditions provided by EquiTest System platform (NeuroCom, Clackamas, OR) (Table 1). During testing, subjects stood on a force plate with their feet positioned shoulder width apart and their arms folded across their chest. They were instructed to maintain stable upright posture for 20 s. The participants performed all 7 of the SOTs conditions and repeated each trial 3 times. Each participant completed the testing in the standardized order as shown in Table 1.

**TABLE 1:** Sensory organization tests (SOTs) used for testing balance control. The sway referenced surrounding is used to disrupt visual feedback. The sway referenced platform is used to disrupt somatosensory feedback. SOT-2M and SOT-5M tests increase the sensitivity of SOT-2 and SOT-5 by requiring subjects to pitch their heads ±20° at 0.33 Hz as cued by an oscillating tone provided over headphones N/A: Not applicable.

Test	Eyes	Surroundings	Platform	Head position
SOT-1	Open	Fixed	Fixed	Upright
SOT-2	Closed	N/A	Fixed	Upright
SOT-3	Open	Sway referenced	Fixed	Upright
SOT-4	Open	Sway referenced	Sway referenced	Upright
SOT-5	Closed	N/A	Sway referenced	Upright
SOT-2M	Closed	N/A	Fixed	Head pitch ±20°
SOT-5M	Closed	N/A	Sway referenced	Head pitch ±20°

The subjects wore headphones with Inertial sensors (Xbus Kit; Xsens Technologies B. V., Enschede, Netherlands) mounted on them that were used to quantify head position. Trials during which subjects were unable to perform the head movements appropriately (defined as two standard deviations from the mean for either amplitude or frequency) were removed. Trials were terminated if the subjects moved their feet, began to take a step, or raised their arms (Wood et al., 2015).

The center of pressure was calculated from the data obtained from the force plate sampled at 100 Hz and then filtered to estimate center of mass (COM). The subjects’ sway angles were then derived from the COM that was assumed to be above the support surface at approximately 55% of the subject height (SMART EquiTest System Operator’s Manual, NeuroCom International). The anterior-posterior (AP) peak-to-peak sway angle of the COM was used to compute a continuous equilibrium (EQ) score scaled relative to a maximum theoretical peak-to-peak sway of 12.5° and further normalized by the percentage of the trial time that the subject completed (Wood et al., 2012). The primary dependent variable in this assessment was the median of the EQ scores across the 3 trails, as has been reported previously (Wood et al., 2011).

### Statistical analysis

The measurements obtained from the multiple trials were averaged. Statistical analyses were performed using Matlab (R Core Team, 2023). Non-parametric tests were used because multiple measures obtained before and after bed rest were not normally distributed. The Kruskal-Wallis with *post hoc* Mann Whitney U-Test was performed to examine differences between groups using measures obtained before bed rest. A linear mixed-effects model was fit using measures from each of the 4 subject groups at 2 time points (before and after bed rest). Analysis of variance F-tests were performed for each of the fixed-effects terms in the linear mixed-effects model. Based on those results, *post hoc* Wilcoxon Signed-Rank tests were performed to examine changes pre- to post-bed rest for each group. The Tukey-Kramer *P*-value correction was used for multiple comparisons. The alpha level of significance for all analyses was set at 0.05.

## Results

**FIGURE 2:**
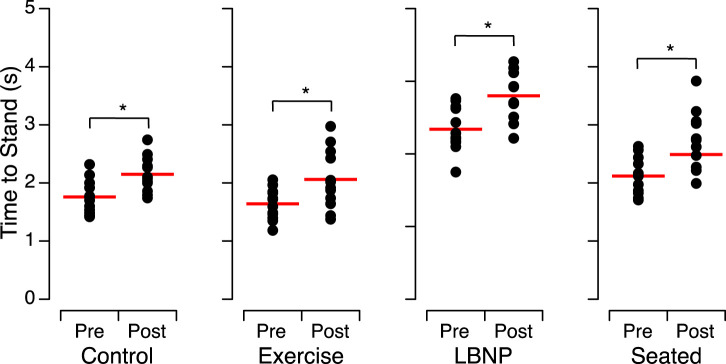
Time to stand from a sitting position and maintain a stable posture before (Pre) and after (Post) head-down tilt bed rest for the individuals of each group. The closed symbols show the means of 2 trials for each subject and the horizontal red bars show the means of all participants. *p < 0.05.

Before HDT bed rest, the time required to stand from a sitting position during the sit-to-stand test was significantly different between the subject groups (Table 2). All 4 subject groups took significantly longer [average relative percent change (range) = 24.1% (19.3%–29.0%)] to stand after HDT bed rest than they did before bed rest (Figure 2; Table 3).

**TABLE 2:** First row: Kruskal-Wallis *p*-values for outcome measures that were significantly different between groups pre-HDT. Other rows: *Post-hoc* Mann Whitney U-Test *p*-values for significant group comparisons. ns, not significative.

	Sit-to-stand (time to stand)	Walk-and-turn (time to complete)	Walk-and-turn (turn rate)
Between group pre-HDT *p*-values	<0.001	<0.001	0.001
Control vs. LBNP	0.003	<0.001	ns
Control vs. seated	ns	0.003	ns
Exercise vs. LBNP	<0.001	<0.001	0.003
Exercise vs. seated	0.02	0.001	0.013

**TABLE 3:** Mean ± standard deviation and *p*-values from the *post hoc* Wilcoxon Signed-Rank tests comparing the changes before and after HDT bed rest for each subject group. The Tukey-Kramer *p*-value correction was used for multiple comparisons. The alpha level of significance for all analyses was set at 0.05.

Task	Outcome	Control			Exercise			LBNP			Seated		
		Pre-HDT	Post-HDT	*p*-value	Pre-HDT	Post-HDT	*p*-value	Pre-HDT	Post-HDT	*p*-value	Pre-HDT	Post-HDT	*p*-value
Sit-to-stand	Time to stand (s)	1.76 ± 0.29	2.15 ± 0.30	0.003	1.64 ± 0.27	2.06 ± 0.51	0.004	2.34 ± 0.30	2.80 ± 0.35	0.019	2.12 ± 0.33	2.74 ± 0.51	0.004
Walk-and-turn	Time to complete (s)	9.08 ± 1.11	10.8 ± 2.01	0.020	8.91 ± 0.97	10.2 ± 1.75	0.009	12.6 ± 1.81	14.75 ± 3.05	0.001	12.0 ± 1.42	14.4 ± 3.65	0.009
Walk-and-turn	Turn rate (deg/s)	115 ± 15.9	98.0 ± 16.6	0.001	128 ± 19.2	107 ± 17.4	0.020	97.5 ± 18.7	86.0 ± 16.6	0.009	102 ± 14.2	93.8 ± 20.7	**0.083**
Tandem walk	% steps, eyes closed	85.3 ± 11.8	65.6 ± 17.3	0.006	83.9 ± 15.5	62.6 ± 22.7	0.008	83.2 ± 17.0	53.3 ± 16.4	<0.001	85.6 ± 15.1	76.8 ± 10.4	0.044
Tandem walk	% steps, eyes open	99.2 ± 1.92	89.6 ± 9.00	0.001	98.5 ± 2.80	87.9 ± 11.0	0.013	97.19 ± 5.14	86.3 ± 13.8	0.007	95.8 ± 6.97	88.2 ± 10.4	0.003
SOT-1	EQ score (%)	91.0 ± 3.86	88.2 ± 4.90	0.020	87.9 ± 6.31	90.0 ± 2.34	**0.464**	89.5 ± 4.65	87.9 ± 5.66	**0.677**	86.6 ± 6.30	87.5 ± 7.45	**0.898**
SOT-2	EQ score (%)	92.7 ± 2.01	88.4 ± 4.27	0.009	90.5 ± 3.19	87.8 ± 4.13	0.053	92.1 ± 2.24	87.4 ± 3.68	0.002	91.8 ± 3.80	87.4 ± 5.24	**0.083**
SOT-3	EQ score (%)	92.2 ± 2.59	88.6 ± 5.43	0.012	91.3 ± 2.35	90.7 ± 3.08	**0.831**	91.5 ± 3.81	87.7 ± 4.79	0.042	88.5 ± 8.33	88.8 ± 5.44	**0.519**
SOT-4	EQ score (%)	82.0 ± 6.60	74.2 ± 10.4	0.042	81.0 ± 9.77	74.3 ± 14.9	0.041	80.6 ± 8.80	74.3 ± 9.83	**0.052**	77.5 ± 12.2	80.2 ± 6.92	**0.764**
SOT-5	EQ score (%)	69.9 ± 6.96	61.4 ± 11.4	0.009	63.7 ± 11.4	57.7 ± 13.5	**0.206**	64.8 ± 11.6	57.3 ± 14.7	**0.151**	69.3 ± 7.22	67.6 ± 8.58	**0.464**
SOT-2M	EQ score (%)	91.9 ± 1.88	79.9 ± 6.79	<0.001	92.0 ± 2.41	80.3 ± 10.2	<0.001	91.2 ± 3.44	75.9 ± 12.2	0.001	87.4 ± 6.83	84.0 ± 6.23	**0.365**
SOT-5M	EQ score (%)	53.1 ± 6.33	47.1 ± 13.0	**0.206**	53.1 ± 11.7	41.7 ± 12.9	0.041	45.5 ± 14.5	37.3 ± 20.7	**0.16**	51.4 ± 11.3	51.6 ± 9.35	**0.898**

Bold represents *p*-value > 0.05.

The time required to complete the walk-and-turn tests before HDT bed rest was significantly different between the subject groups (Table 2). After HDT bed rest, all the subject groups took significantly longer to complete the walk-and-turn task than they did before HDT bed rest, with an average percent change of 17.6% [14.4%–19.8%] slower than post-HDT bed rest (Table 3).

**FIGURE 3:**
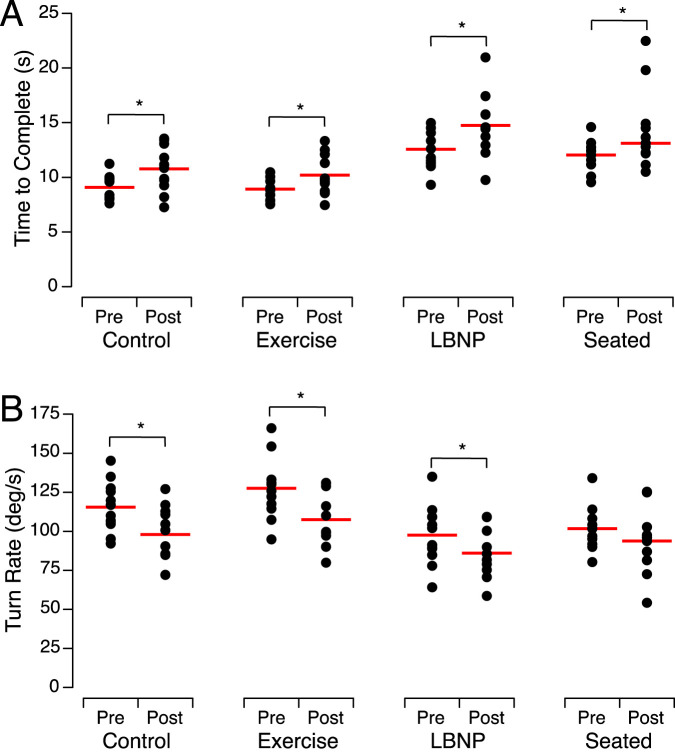
Time to complete the walk-and-turn task **(A)** and turn rate around the cone **(B)** before (Pre) after (Post) head-down tilt bed rest for the individuals of each group. The closed symbols show the means of 2 trials for each subject and the horizontal red bars show the means of all participants. *p < 0.05.

Before HDT bed rest, the turn rate around the cone during the walk-and-turn task was significantly different between the subject groups (Table 2). After HDT bed rest, subjects of the control group, the exercise group, and the LBNP group turned slower around the cone at mid-course than they did before HDT bed rest, with an average absolute percent change of 14.2% [11.8%–15.8%]. By contrast, subjects of the seated group were 7.7% slower after HDT bed rest compared to pre-HDT (Figure 3; Table 3).

**FIGURE 4:**
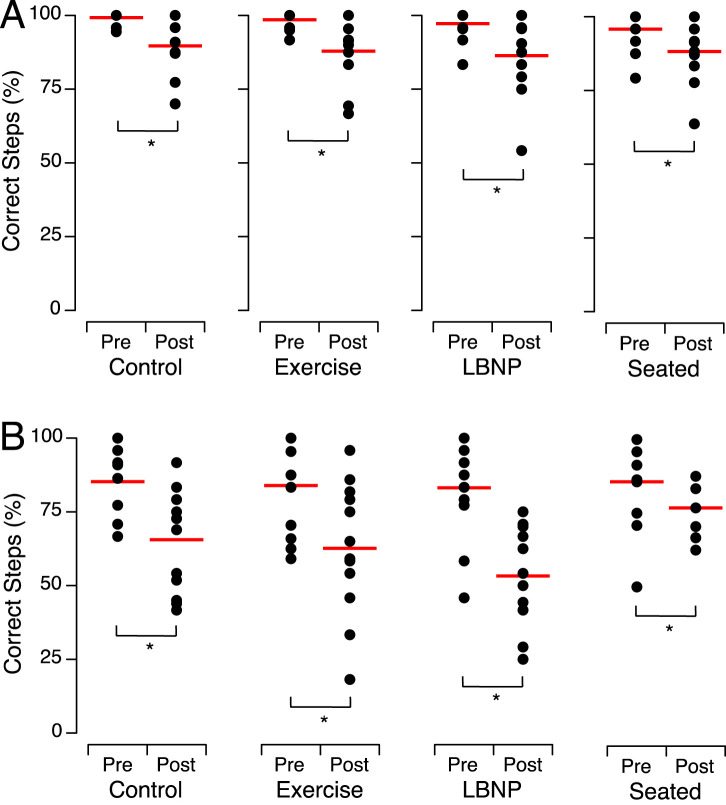
Percent of correct steps during tandem walk with the eyes open **(A)** and the eyes closed **(B)** before (Pre) and after (Post) head-down tilt bed rest for the individuals of each group. The closed symbols show the means of 2 trials for each subject and the horizontal red bars show the means of all participants. *p < 0.05.

Before HDT bed rest, no significant difference was detected in each subject groups’ percentage of correct steps while they performed the tandem walk tests, either with their eyes open or with their eyes closed. After HDT bed rest, all 4 subject groups had significantly lower percentages of correct steps, both with their eyes open and their eyes closed, than they did before bed rest (Figure 4; Table 4). The average absolute relative percent change between pre- and post-HDT was 23.7% [10.3%–36.0%] for eyes closed and 9.9% [7.9%–11.2%] for eyes open.

**TABLE 4:** Results of the linear mixed model for the outcomes of the functional tasks before and after 30-day HDT bed rest (Group: control, exercise, LBNP, seated; Time: before HDT, after HDT).

Task	Term	F	df1	df2	*p* value
Sit-to-stand (time to stand)	Intercept	302.58	1	84	<0.001
	Group	10.339	3	84	<0.001
	Time	9.166	1	84	0.003
	Group × Time	0.564	3	84	0.639
Walk-and-turn (time to complete)	Intercept	242.94	1	84	<0.001
	Group	10.73	3	84	<0.001
	Time	8.649	1	84	0.004
	Group × Time	0.741	3	84	0.530
Walk-and-turn (turn rate)	Intercept	567.51	1	84	<0.001
	Group	7.875	3	84	<0.001
	Time	11.977	1	84	<0.001
	Group × Time	1.091	3	84	0.357
Tandem walk, eyes open (time to complete)	Intercept	1776.2	1	85	<0.001
	Group	0.393	3	85	0.764
	Time	10.095	1	85	0.002
	Group × Time	0.225	3	85	0.878
Tandem walk, eyes closed (time to complete)	Intercept	326.48	1	84	<0.001
	Group	0.049	3	84	0.980
	Time	16.848	1	84	<0.001
	Group × Time	3.436	3	84	0.020

**FIGURE 5:**
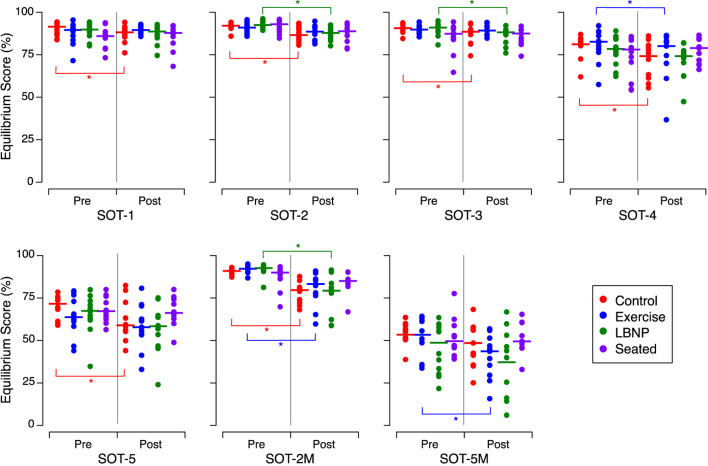
Equilibrium scores during the 7 sensory organization tests (SOT) before (Pre) and after (Post) head-down tilt bed rest for the individuals of each group. The symbols show the mean of the 3 trials for the individuals in each subject group. *p < 0.05. The *P*-values of *post hoc* tests are detailed in the Supplementary Material.

Before HDT bed rest, no significant differences were detected in EQ scores between the subject groups for any of the 7 SOT conditions. After HDT bed rest, the control subjects exhibited markedly reduced performance in the dynamic posturography test, as indicated by EQ scores, across all SOT conditions, except during pitch head movements on the sway-referenced platform (SOT-5M) (Figure 5; Table 5). After HDT bed rest, the subjects who performed the exercise protocol displayed a significant decline in EQ scores for the SOT-4, SOT-2M, and SOT-5M conditions compared to their scores before HDT bed rest. Individuals who underwent LBNP demonstrated a notable decrease in EQ scores for the SOT-2, SOT-3, and SOT-2M conditions after HDT bed rest compared to their scores before HDT bed rest. The EQ scores for the subjects who sat intermittently during the HDT phase were not significantly different before and after bed rest for all the SOT conditions (Figure 5; Table 5).

**TABLE 5:** Results of the linear mixed model for the equilibrium score during the dynamic posturography task before and after 30-day HDT bed rest (Group: control, exercise, LBNP, seated; Time: before HDT, after HDT).

SOT	Term	F	df1	df2	*p-value*
SOT-1	Intercept	3734.4	1	86	<0.001
	Group	1.661	3	86	0.181
	Time	1.819	1	86	0.180
	Group × Time	1.123	3	86	0.344
SOT-2	Intercept	8327.1	1	86	<0.001
	Group	0.854	3	86	0.467
	Time	10.191	1	86	0.002
	Group × Time	0.439	3	86	0.725
SOT-3	Intercept	4734.7	1	86	<0.001
	Group	1.497	3	86	0.221
	Time	3.671	1	86	0.058
	Group × Time	1.212	3	86	0.310
SOT-4	Intercept	849.13	1	86	<0.001
	Group	0.478	3	86	0.698
	Time	6.533	1	86	0.012
	Group × Time	2.378	3	86	0.075
SOT-5	Intercept	530.43	1	86	<0.001
	Group	1.060	3	86	0.370
	Time	5.475	1	86	0.021
	Group × Time	0.624	3	86	0.600
SOT-2M	Intercept	2330.9	1	83	<0.001
	Group	1.303	3	83	0.278
	Time	20.926	1	83	<0.001
	Group × Time	3.648	3	83	0.015
SOT-5M	Intercept	224.65	1	83	<0.001
	Group	1.041	3	83	0.378
	Time	2.863	1	83	0.152
	Group × Time	1.397	3	83	0.249

**FIGURE 6:**
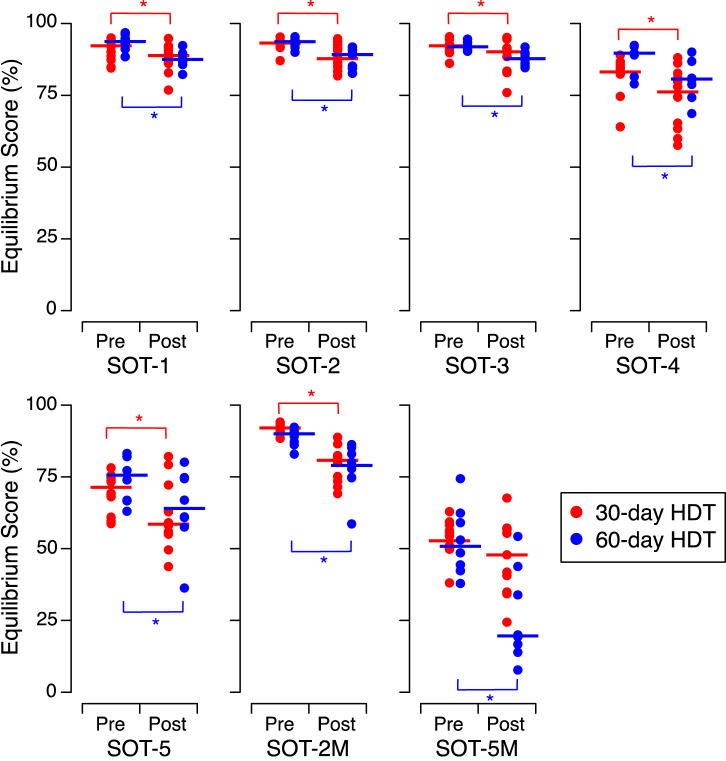
Equilibrium scores during the 7 sensory organization tests (SOT) before (Pre) and after (Post) head-down tilt bed rest for the control subjects in the present SANS-CM study (30-day HDT, N = 12) and the control subjects during the previous AGBRESA study (60-day HDT, N = 8). The symbols show the means of the 3 trials for the individuals in each subject group. *p < 0.05. The *p*-values of *post hoc* tests are detailed in the Supplementary Material.

We compared the alterations in EQ scores among the control subjects in the present study, which included 12 participants undergoing 30 days of HDT, with those observed in eight control subjects after a previous HDT bed rest lasting 60 days (Clément et al., 2022b). Prior to HDT bed rest, the EQ scores were significantly different between the subjects of both studies only for SOT-4 (Mann Whitney, *p* = 0.041). After 60 days of HDT bed rest, the control subjects exhibited a significant decrease In EQ scores in the SOT-5M condition (Figure 6; Table 6). This finding differs from our current study, as we observed no significant decrease in EQ scores under the SOT-5M condition following 30 days of HDT bed rest (see Figure 5; Supplementary Table S1).

**TABLE 6:** Results of the linear mixed model for the equilibrium score before and after 30-day and 60-day HDT bed rests (Group: 30 days, 60 days; Time: before HDT, after HDT).

SOT	Term	F	df1	df2	*p* value
SOT-1	Intercept	5068.7	1	36	<0.001
	Group	3.309	1	36	0.077
	Time	9.361	1	36	0.004
	Group × Time	1.312	1	36	0.259
SOT-2	Intercept	7,946	1	36	<0.001
	Group	<0.001	1	36	0.996
	Time	13.938	1	36	<0.001
	Group × Time	0.383	1	36	0.539
SOT-3	Intercept	5993.2	1	36	<0.001
	Group	0.011	1	36	0.913
	Time	5.994	1	36	0.019
	Group × Time	0.052	1	36	0.819
SOT-4	Intercept	1165.7	1	36	<0.001
	Group	3.329	1	36	0.076
	Time	5.013	1	36	0.031
	Group × Time	0.003	1	36	0.956
SOT-5	Intercept	493.48	1	36	<0.001
	Group	0.629	1	36	0.432
	Time	8.567	1	36	0.005
	Group × Time	0.162	1	36	0.689
SOT-2M	Intercept	2231.9	1	35	<0.001
	Group	1.141	1	35	0.292
	Time	17.779	1	35	<0.001
	Group × Time	0.051	1	35	0.821
SOT-5M	Intercept	176.73	1	35	<0.001
	Group	<0.001	1	35	0.978
	Time	22.191	1	35	<0.001
	Group × Time	7.850	1	35	0.008

## Discussion

The results of the current study indicate that 30 days of axial unloading during HDT bed rest degraded performance of the functional tasks that require body coordination and postural stability control. While the interventions applicable during spaceflight (exercise, cuffs, LBNP) mitigate this performance degradation, they are not as effective as spending 6 h per day in an upright position without any other countermeasures, a condition that is rather challenging to achieve in orbit. Furthermore, sitting upright was only effective for some of the measures in this study, namely the turn rate during the walk-and-turn task and the equilibrium scores during dynamic posturography SOTs. A more direct, active sensorimotor countermeasure is likely required for better protection against functional task performance decrements after HDT bed rest.

All subject groups required more time to stabilize themselves during the sit-to-stand test after bed rest. Likewise, all subject groups took more time after bed rest to complete the walk-and-turn test. It has been proposed that the delay in rising from a seated position after HDT bed rest could be attributed to decreased blood pressure, because changes in postural orientation induce an orthostatic challenge, muscle weakness, alteration in neuromuscular coordination, poor balance, and fear of falling (Mulavara et al., 2018).

The dynamic posturography test is primarily designed to assess the effectiveness of vestibular and somatosensory inputs in maintaining balance during quiet stance. Substantial declines in performance of this test have been noted both after spaceflight and after bed rest (Reschke et al., 2009; Wood et al., 2015; Reschke and Paloski, 2017; Mulavara et al., 2018; Miller et al., 2018; Clément et al., 2022a). Theoretically, prolonged exposure to HDT should not impact signaling from the semicircular canals or the otoliths. However, during bed rest, the body undergoes axial unloading despite the presence of gravity, which diminishes inputs to somatosensory receptors distributed throughout the body. Proprioception and body-load sensors play a crucial role in controlling gait and maintaining postural stability (Reschke et al., 2009). It has been suggested that the unloading of the body and subsequent loss of support afferentation experienced during spaceflight triggers a series of neuromotor changes leading to neuromuscular dysfunction, including decreased activation of tonic muscle and subsequent instability in posture and locomotion after spaceflight (Kozlovskaya et al., 2007).

The aim of the tandem walk test was to evaluate bed rest-induced alterations in control of dynamic balance. This test is a highly valid measure of vestibular-driven ataxia (Fregly et al., 1972). Previous studies found that subjects who did not exercise during bed rest showed declines in tandem walk performance after 60–70 days of HDT bed rest (Macaulay et al., 2016; Miller et al., 2018). In our investigation, which employed a 30-day HDT protocol, all subjects’ performance in a tandem walk test were reduced after bed rest, even the performance of those subjects who exercised during the HDT phase, although the exercise was limited to aerobic cycling and did not include resistive training.

Previous studies have demonstrated that HDT bed rest induces significant impairment in dynamic balance control, even when subjects engaged in a variety of resistive and cardiovascular exercises during the HDT phase (Dupui et al., 1992; Mulavara et al., 2018). Similarly, in our study, we observed that exercise with subsequent donning of thigh cuffs or that exposure to LBNP did not ameliorate the declines in tandem walk performance and EQ scores after HDT bed rest. It is evident that cardiovascular exercise and passive sequestering of blood in the legs by means of thigh cuffs or LBNP were insufficient to counteract the HDT bed rest-induced effects on control of dynamic balance.

Changes in muscle stiffness, length, strength, and composition have been observed during prolonged bed rest and could also contribute to the alterations in dynamic balance control. Biopsies have shown a shift in muscle fiber composition from slow-twitch (Type I) fibers, which are more resistant to fatigue, to fast-twitch (Type II) fibers, which, although less resistant to fatigue, can generate more force quickly and are functionally relevant in recovering from a trip or loss of balance (Behan et al., 2018). Increased collagen deposition in the muscles and tendons leads to greater stiffness and reduced elasticity, thereby affecting movement efficiency (Hargens and Vico, 2016; Schoenrock et al., 2018).

However, adopting an intermittent upright seated posture (2 h × 3 h) during the HDT phase proved beneficial in mitigating the decline in tandem walk performance after bed rest. Additionally, it helped counteract the post-HDT bed rest reduction in EQ scores observed during the dynamic posturography test in conditions SOT-2, SOT-3, SOT-4, and SOT-5, where visual and somatosensory inputs were altered. These findings underscore the significance of proprioceptive and skin receptors in maintaining balance control and suggest that alterations in the central processing of information from muscle spindles and skin receptors greatly contribute to control of dynamic balance after axially unloading of the body.

Neurophysiological investigations support the concept that when individuals receive unreliable vestibular information, they depend more on Supplementary Material such as proprioception to uphold their posture and control their movements (Yates et al., 2000; Carriot et al., 2015). Additionally, it has been established that the vestibular nuclei integrate data from various sensory inputs, including the labyrinth, neck, spinal cord, and the limbs (Jian et al., 2002). Upregulation of vestibular inputs during bed rest are thought to be a response to the reduced somatosensory inputs (Yuan et al., 2018). Consequently, we deduce that the changes in performance during the dynamic posturography and tandem walk tests after bed rest reflect deconditioning in somatosensory functions (Mulavara et al., 2018).

Prolonged exposure to microgravity during spaceflight significantly impacts both static and dynamic balance (Clément et al., 2022a). In microgravity, vestibular, proprioceptive, and visual cues differ substantially from those experienced on Earth, disrupting perception of space and control of movement (Clément et al., 2020). Muscle atrophy and weakness in the balance-maintaining muscles can occur during extended exposure to microgravity, leading to reduced stability and control after return to Earth. After returning to Earth, astronauts experience temporary instability due to the microgravity-induced readjustment of sensory inputs, causing difficulties with balance and coordination (Wood et al., 2011). Astronauts can also experience long-term alterations in their balance and gait after spaceflight that can impact their ability to perform tasks requiring precise balance control. Understanding these effects is vital for developing strategies to maintain astronaut health and performance during space expeditions, particularly in surface exploration missions where bulky suits are worn in a gravity-altered environment (Macaulay et al., 2021).

Our results indicate that sitting upright for 6 h a day during HDT bed rest has beneficial effects on both static and dynamic balance. However, astronauts feel weightless in space because they are in a constant state of free fall around Earth, and there is no solid surface or external force opposing this motion. Researchers have explored the use of artificial gravity, typically through centrifugation, as a measure to counteract the negative effects of HDT bed rest. Centrifugation involves rotating the bed rest subjects in a circular motion, creating a centrifugal force that simulates gravity. Exposing individuals to this artificial gravity for 30 min to 1 h per day during bed rest studies has demonstrated potential for safeguarding muscle strength, bone density, cardiovascular function, and various other physiological parameters (Clément et al., 2015). However, this intermittent artificial gravity set at 1 g at the center of mass (and 2 g at the feet) did not maintain balance and functional mobility after bed rest (Mulder et al., 2014; Reschke and Paloski, 2017; Tays et al., 2022; Clément et al., 2022b). The durations of 30 min to 1 h of artificial gravity exposure were selected to mirror the duration of exercise performed by astronauts on board the International Space Station. Our study findings indicate that exposure to 1 g gravity for several hours may be more beneficial to counter the bed rest-induced effects on mobility and balance. Further research is required to refine the optimal duration and intensity of artificial gravity to potentially serve as a countermeasure for preserving functional task performance and balance of bed rest subjects and astronauts.

The countermeasures evaluated and discussed thus far have important effects on headward fluid shifts, cardiovascular functions, and proprioceptive inputs. However, it is likely that more direct, active sensorimotor countermeasures are needed to fully mitigate declines in functional task performance during bed rest. High intensity resistance and aerobic exercise is a proven approach to provide the axial loading, tactile input, and exercise stimulus to maintain muscular functions and accelerate sensorimotor recovery; however, this countermeasure also does not fully protect postural and locomotor control after bed rest (Miller et al., 2018). The limited effects of such exercise countermeasures on upright function may be due to the focus on motor output, without incorporating the sensory aspects of postural control (Aman et al., 2015; Schoenrock et al., 2018; English et al., 2020). Countermeasures may need to target the proprioceptive and tactile systems in the context of dynamic postural challenges during bed rest to keep the proprioceptive and tactile systems conditioned and adaptable to upright postural challenges (Macaulay et al., 2021). Such countermeasures should be evaluated in future bed rest and spaceflight studies.

## Data Availability

The datasets presented in this study can be found in online repositories. The names of the repository/repositories and accession number(s) can be found below: NASA Life Science Data Portal.
