# Supplementary material for: Effects of intermittent seating upright, lower body negative pressure, and exercise on functional tasks performance after head-down tilt bed rest
Source: Front Physiol. 2024 Sep 6;15:1442239. doi: 10.3389/fphys.2024.1442239 (PMC11412840; doi:10.3389/fphys.2024.1442239)
Supplement: Supplementary file 1 [file Table1.DOCX]

**Supplementary material**

**Table S1**. *P*-values from changes in the equilibrium score (EQ Score) before and after HDT best rest for the control subjects in the present SANS-CM study (30 days, N=12) and for the control subjects in the AGBRESA study of longer duration (60 days, N=8). The Tukey-Kramer *p*-value correction was used for multiple comparisons. The alpha level of significance for all analyses was set at 0.05.

| **Task** | **Outcome** | **30 days** | **60 days** |
| --- | --- | --- | --- |
| SOT-1 | EQ Score | 0.021 | 0.008 |
| SOT-2 | EQ Score | 0.009 | 0.008 |
| SOT-3 | EQ Score | 0.012 | 0.008 |
| SOT-4 | EQ Score | 0.042 | 0.023 |
| SOT-5 | EQ Score | 0.009 | 0.008 |
| SOT-2M | EQ Score | <0.001 | 0.008 |
| SOT-5M | EQ Score | 0.206 | 0.039 |
